# Association of Gut Microbiota Enterotypes with Blood Trace Elements in Women with Infertility

**DOI:** 10.3390/nu14153195

**Published:** 2022-08-04

**Authors:** Xinrui Yao, Na Zuo, Wenzheng Guan, Lingjie Fu, Shuyi Jiang, Jiao Jiao, Xiuxia Wang

**Affiliations:** 1 Center of Reproductive Medicine, Shengjing Hospital of China Medical University, 39 Huaxiang Road, Shenyang 110004, China; 2 Shenyang Reproductive Health Clinical Medicine Research Center, Shenyang 110004, China

**Keywords:** enterotypes, whole blood trace elements, infertility, iron metabolism

## Abstract

Infertility is defined as failure to achieve pregnancy within 12 months of unprotected intercourse in women. Trace elements, a kind of micronutrient that is very important to female reproductive function, are affected by intestinal absorption, which is regulated by gut microbiota. Enterotype is the classification of an intestinal microbiome based on its characteristics. Whether or not *Prevotella*-enterotype and *Bacteroides*-enterotype are associated with blood trace elements among infertile women remains unclear. The study aimed to explore the relationship between five main whole blood trace elements and these two enterotypes in women with infertility. This retrospective cross-sectional study recruited 651 Chinese women. Whole blood copper, zinc, calcium, magnesium, and iron levels were measured. Quantitative real-time PCR was performed on all fecal samples. Patients were categorized according to whole blood trace elements (low levels group, <5th percentile; normal levels group, 5th‒95th percentile; high levels group, >95th percentile). There were no significant differences in trace elements between the two enterotypes within the control population, while in infertile participants, copper (*P* = 0.033), zinc (*P* < 0.001), magnesium (*P* < 0.001), and iron (*P* < 0.001) in *Prevotella*-enterotype was significantly lower than in *Bacteroides*-enterotype. The Chi-square test showed that only the iron group had a significant difference in the two enterotypes (*P* = 0.001). Among infertile patients, *Prevotella*-enterotype (Log(*P/B*) > −0.27) predicted the low levels of whole blood iron in the obesity population (AUC = 0.894; *P* = 0.042). For the high levels of iron, *Bacteroides*-enterotype (Log(*P/B*) <−2.76) had a predictive power in the lean/normal group (AUC = 0.648; *P* = 0.041) and Log(*P/B*) <−3.99 in the overweight group (AUC = 0.863; *P* = 0.013). We can infer that these two enterotypes may have an effect on the iron metabolism in patients with infertility, highlighting the importance of further research into the interaction between enterotypes and trace elements in reproductive function.

## 1. Introduction

Infertility, which is commonly defined as no pregnancy after one year of unprotected intercourse, affects millions of couples worldwide [1]. Gut microbiota, a complex community of microorganisms living in the intestinal tract of humans and animals, is very diverse in species [2] and genes [3]. Recently, studies have linked the relationship between gut microbiota and endocrine and metabolism disorders, especially with diseases of the female reproductive endocrine system [4]. Imbalances in the gut microbiota composition can impair women’s reproductive function and cause related diseases and conditions such as polycystic ovary syndrome (PCOS), endometriosis, pregnancy complications, and adverse pregnancy outcomes [5]. However, the specific mechanisms of gut microbiota affecting female reproductive function are still limited.

Trace elements are essential in basic metabolic processes such as enzymatic reactions, playing an indispensable role in the human body, and an appropriate amount of some trace elements such as copper, zinc, calcium, magnesium, and iron on well-being is essential, especially for the reproductive function of women [6,7]. For example, there is considerable evidence in female reproductive systems highlighting zinc effects on oocyte development and maturation, egg activation, and ovarian function [8]. In peripheral tissues, iron serves as a cofactor for the expression and activation of various metabolic enzymes involved in glycolysis, electron transfer chain, and the TCA cycle, which is essential for follicle development [9]. The deficiency of these micronutrients can reduce fertility and cause unfavorable pregnancy outcomes [10]. Carl et al. concluded that maternal copper deficiency can lead to intrauterine growth retardation, teratogenicity, fetal death, and persistent postpartum complications [11]. Dietary zinc deficiency reduces oocyte quality; hence, adequate zinc is necessary for oocytes to develop into fertilized eggs [8]. Calcium deficiency during pregnancy affects epigenetic regulation of gene expression and induces various metabolic phenotypes in their offspring, such as insulin resistance [12]. Experiments with mice revealed that magnesium deficiency during pregnancy may adversely affect the placental function and fetal weight [13]. Iron deficiency is associated with adverse pregnancy outcomes, including increased maternal disease, preterm birth, intrauterine growth restriction, and low birth weight [14].

The key role that gut microbiota plays in human health has inspired research to identify microbes and their functions associated with metabolic pathways, particularly those associated with the metabolism of dietary components [15]. Recent work has demonstrated that microorganisms and microbial genes (the gut microbiome) can regulate the metabolism and transport of micronutrients in the human body; in turn, they can increase the bioavailability of trace elements by influencing food assimilation or competing with the hosts [16]. However, the gut microbiome of different individuals varies greatly on a time and space scale [17], which increases the difficulties and obstacles in the medical research and application of gut microbes. Through the analysis of human microbiome genomes from 39 samples in six nationalities, Arumugam et al. [18] first introduced the concept of “enterotypes.” The gut microbiome of different individuals can be divided into two enterotypes according to the dominant bacteria genera. *Prevotella*-enterotype and *Bacteroides-*enterotype are the two dominant enterotypes in the human gut [19], and the relative abundance of *Prevotella* divided by *Bacteroides* (*P/B* ratio) can be used to stratify these two enterotypes [20,21,22]. The enterotypes are relatively stable, mainly according to long-term dietary habits, and has no direct relationship with gender, age, geography, and cultural background [18]. Besides external factors, the relationship between enterotypes and intrinsic factors such as host genetic and immune factors is unclear [23]. There are distinctive different digestive functions between these two enterotypes; *Prevotella*-enterotype can effectively hydrolyze plant fiber and has the fermentation potential of low fat and low protein. On the contrary, *Bacteroides-*enterotype has specific digestive enzymes that degrade animal carbohydrates and are also efficient in digesting proteins [24,25]. Patients with *Prevotella*-enterotype and *Bacteroides-*enterotype also respond differently to dietary fiber [26,27]. These results indicate the important role of two enterotypes in nutrient metabolism.

Despite these advances in knowledge, a limited number of studies have related microbial enterotypes to trace elements, especially for women with reproduction problems. Hence, we aimed to explore the relationship between enterotypes and the status of five main whole blood trace elements (copper, zinc, calcium, magnesium, and iron) in infertile women.

## 2. Materials and Methods

### 2.1. Study Design and Study Population

**Figure 1:**
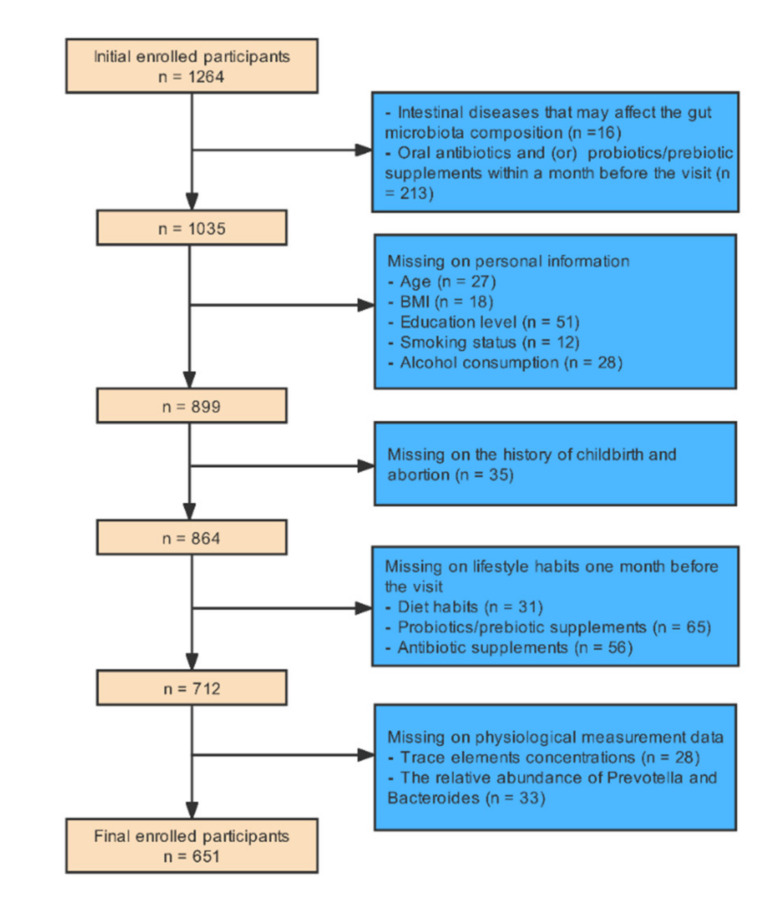
The exclusion procedure of study population.

This single-center, retrospective, cross-sectional study was conducted on a sample of 1264 women pursuing medical treatment at the center of reproductive medicine, Shengjing Hospital of China Medical University from September 2020 to September 2021. This study mainly used the methods of medical physiological measurement and questionnaire survey. The inclusion criteria for infertile patients were non-male infertility and inability to conceive a child within 12 months of regular sexual activity without using any contraceptive methods [28]. The inclusion criteria for participants in the control group were those with regular menstrual cycles, normal ovarian morphology, and normal hormone levels unable to conceive because of their partners’ reasons. Exclusion criteria were participants with a history of major gastrointestinal illness that may affect the gut microbiota (n = 16) and participants who had taken antibiotics and (or) probiotics/prebiotic supplements within a month before the visit (n = 213). Each participant was asked to complete a paper questionnaire containing some questions, which could be divided into three parts: personal information, childbirth and abortion history, and lifestyle habits for approximately a month. We also excluded participants with missing information on five main whole blood trace elements (copper, zinc, calcium, magnesium, and iron) concentrations (n = 28), the relative abundance of *Prevotella* and *Bacteroides* (n = 33), age (n = 27), BMI (n = 18), smoking status (n = 12), alcohol consumption (n = 28), education level (n = 51), childbirth and abortion history (n = 35), diet habits in the month prior to the visit (n = 31), probiotics/prebiotic supplements one month prior to the visit (n = 65), and antibiotic supplements one month prior to the visit (n = 56). Finally, 651 participants (182 healthy women, 469 infertile women) were enrolled in our study. Figure 1 presents a detailed description of the exclusion procedure.

The study was conducted in accordance with the Declaration of Helsinki. The study protocol was approved by the Ethics Committee of the Shengjing Hospital of China Medical University (Reference No. 2017PS190K). Informed consent was obtained from all participants.

### 2.2. Sample Measurement

Peripheral venous blood and fecal samples were collected at least 8h after night fasting on the same day during the non-menstrual period. Whole blood copper (Cu), zinc (Zn), calcium (Ca), magnesium (Mg), and iron (Fe) concentrations were determined by flame atomic absorption spectrometry on an atomic absorption spectrometer (BH-5100, Beijing Bohui Innovative Biotechnology Group Corporation Ltd., Beijing, China). Fecal samples were stored after collection at −80 °C until DNA extraction. The TIANamp Fecal DNA Kit (Tiangen Biotechnology (Beijing) Co., Ltd., Beijing, China) was used to extract bacterial DNA from fecal samples according to the manufacturer’s protocol. DNA concentrations were measured with a Qubit 2.0 Fluorometer (Life Technologies, Carlsbad, CA, USA). The integrity of the recovered DNA fragments was determined by agarose gel electrophoresis. Primer sequences for *Prevotella* and *Bacteroides* are listed in Supplementary Material Table S9. The amplification reactions were carried out with 1 μL template (1 ng/μL), 1 μL primer (10 μM each), 0.5 μL probe (10 μM), 12.5 Bestar qPCR Master Mix (DBI-2041, DBI Bioscience, Ludwigshafen, Germany), and 10 μL nuclear-free water in a total volume of 25 μL. Real-Time PCR was performed using the ABI 7500 Real-Time PCR System (Applied Biosystems, Waltham, MA, USA). PCR conditions were as follows: 95 °C for 5 min followed by 40 cycles of 95 °C for 15 s and 56 °C for 40 s. The *Prevotella*-to-*Bacteroides* ratio applies to a general threshold, although the composition of the gut microbiota varies from different regions [23]. The bimodal distributions of the log-normalized *Prevotella*-to-*Bacteroides* (*P/B*) ratio were measured for obtaining a cutoff value, which was used to identify the enterotypes. Height, weight, and BMI were recorded on the day of sampling. According to the Working Group on Obesity in China (WGOC) [29], BMI was categorized as normal/lean, <24 kg/m^2^; overweight, 24–28 kg/m^2^; obesity, >28 kg/m^2^.

### 2.3. Statistical Analysis

The relationship between the two quantitative variables was tested by the Pearson correlation coefficient. The Kolmogorov‒Smirnov test was used to evaluate the normality of the distribution of continuous variables. The Chi-square test and Fisher’s exact test were used to compare categorical variables, and the Kruskal‒Wallis analysis was used to compare continuous variables. Dunnett’s *post-hoc* test (two-sided) was used for multi-group comparisons. Descriptive results are expressed as mean ± standard error (SE) or median (quartile range). The 5th and 95th percentiles were set as the reference values of whole blood trace elements. Whole blood trace elements below the 5th were considered as low level (LL) status, those between the 5th and 95th were considered as normal level (NL) status, and those above the 95th were considered as high level (HL) status. Receiver operating characteristics (ROC) curves were used to test the predictive efficacy of Log(*P/B*) for LL of whole blood trace elements and HL of whole blood trace elements. The area under the ROC curve (AUC) with 95% confidence interval, sensitivity, and specificity were calculated.

Statistical analyses were performed using the Statistical Package for Social Sciences, version 25 (IBM Corp., Armonk, NY, USA). All tests were two-sided, and a *P* value < 0.05 was considered statistically significant.

## 3. Results

### 3.1. Basic Characteristics of the Included Population and the Relationship between the Log(*P/B*), Enterotypes, and Whole Blood Trace Elements

**Figure 2:**
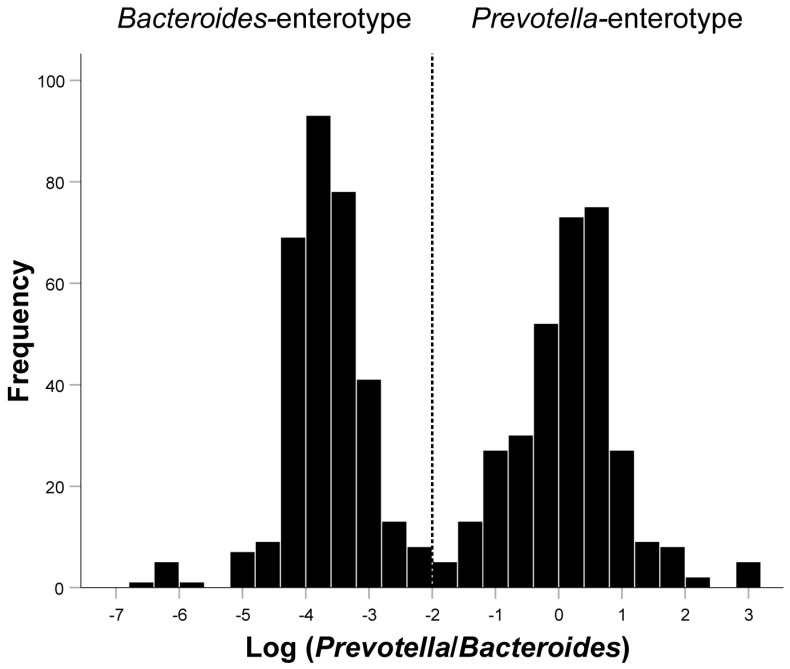
Inferred Log(*P/B*) groups. Fecal distribution of Log(*P/B*) for all patients. Participants were divided into the *Prevotella*-enterotype group and the *Bacteroides*-enterotype group according to the Log(*P/B*) of −2. *Prevotella*-enterotype group, n = 326 (50.08%); *Bacteroides*-enterotype group, n = 325 (49.92%). Log(*P/B*), Log-normalized *Prevotella*-to-*Bacteroides*.

The histogram showed an obvious bimodal distribution when plotting the frequency distribution histogram of Log(*P/B*) ratio (Figure 2). Log(*P/B*) ≥ −2 was considered as *Prevotella*-enterotype, while <−2 was considered as *Bacteroides*-enterotype. As presented in Table 1, the average levels of whole blood zinc (*P* = 0.001), magnesium (*P* < 0.001), and iron (*P* < 0.001) were significantly lower in patients with infertility than in the healthy control group. There were also significant differences between the BMI (*P* = 0.001), Log(*P/B*) (*P* < 0.001), and education levels (*P* < 0.001) between the infertile group and the control group. The trace element levels between the two enterotypes were compared in the infertility group and the control group, respectively. The results showed that only in the infertile group did the whole blood copper (*P* = 0.033), zinc (*P* < 0.001), magnesium (*P* < 0.001), and iron (*P* < 0.001) differ significantly between *Prevotella*-enterotype and *Bacteroides*-enterotype (Table 2). Comparison of five trace elements between two enterotypes under different diseases was shown in Supplementary Material Tables S3–S8.

**Figure 3:**
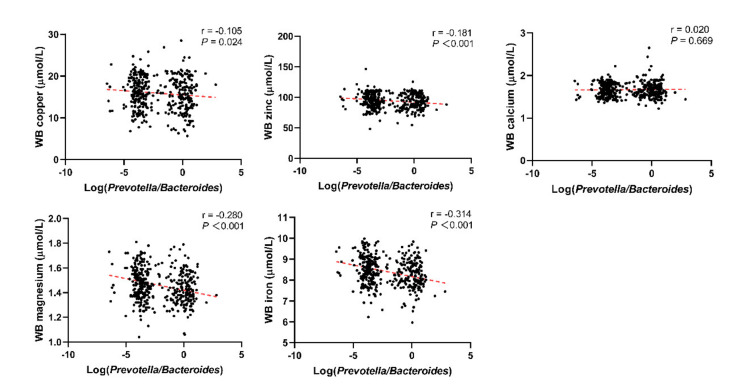
Correlation between Log(*P/B*) and whole blood copper, zinc, calcium, magnesium, and iron levels among infertile group. Log(*P/B*), Log-normalized Prevotella-to-Bacteroides; WB, whole blood.

**Figure 4:**
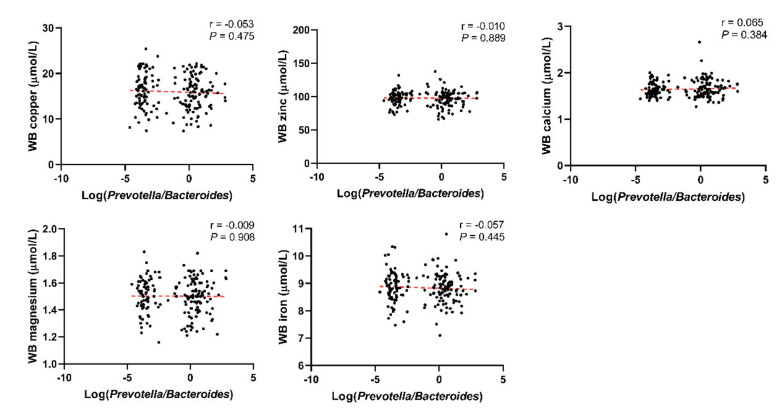
Correlation between Log(*P/B*) and whole blood copper, zinc, calcium, magnesium, and iron levels among control group. Log(*P/B*), Log-normalized Prevotella-to-Bacteroides; WB, whole blood.

Through linear correlation analysis, we observed a negative correlation between Log(*P/B*) and whole blood copper (r = −0.105, *P* = 0.024), zinc (r = −0.181, *P* < 0.001), magnesium (r = −0.280, *P* < 0.001), and iron (r = −0.314, *P* < 0.001) levels (Figure 3). No significant correlation was observed between Log(*P/B*) and these five trace elements in the control population (Figure 4).

Table 3 shows the difference in the enterotypes between the LL, normal levels (NL), and HL of whole blood trace elements in the infertile population. Among the three groups, only the whole blood iron (*P* = 0.001) showed a significant difference in *Prevotella*-enterotype and *Bacteroides*-enterotype. The results of the control group are shown in the Supplementary Material Table S1.

### 3.2. Prevotella-Enterotype Had an Acceptable Predictive Power to Low Levels of Whole Blood Iron in Obese Population

**Figure 5:**
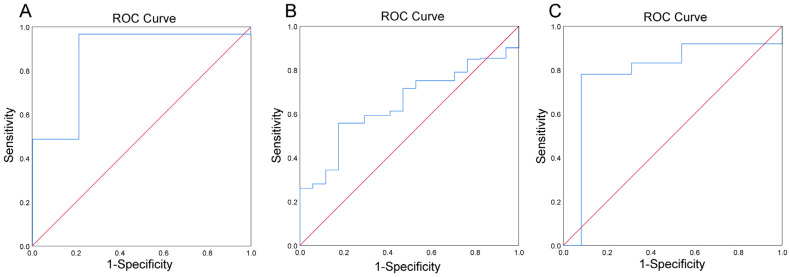
Predictive potential of Log(*P/B*), as estimated using the receiver operating characteristic (ROC) analysis for LL of whole blood iron in obesity group (**A**) HL of whole blood iron in lean/normal group (**B**), and HL of whole blood iron in overweight group (**C**).

Table 4 describes the results of Log(*P/B*), age, BMI, and eating habits for approximately a month between whole blood iron in LL, NL, and HL. Log(*P/B*) was significantly different among the three groups (*P* = 0.002), as well as among the LL and NL groups (*P* = 0.013) and the HL and NL groups (*P* = 0.006). There were significant differences in BMI between the three groups (*P* = 0.045) and between the LL and NL groups (*P* = 0.043), but no significant differences between the HL and NL groups were noted. No significant results were found in the control group (Supplementary Material Table S2). ROC curves evaluated the predictive power of Log(*P/B*) for the LL of whole blood iron and the HL of whole blood iron in lean/normal, overweight, and obese populations. As shown in Table 5 and Figure 5A, Log(*P/B*) (>−0.27) has a predictive power for risk of LL of whole blood iron in the obesity population, with a sensitivity of 98.8% and specificity of 78.8% (AUC = 0.894; *P* = 0.042). For the HL of whole blood iron, when Log(*P/B*) <−2.76, it can predict the HL of whole blood iron in the lean/normal group with a sensitivity of 55.7% and specificity of 82.4% (AUC = 0.648; *P* = 0.041) (Table 6 and Figure 5B). When Log(*P/B*) <−3.99, it can predict the HL of whole blood iron in an overweight group with a sensitivity of 78.1% and a specificity of 92.7% (AUC = 0.863; *P* = 0.013) (Table 6 and Figure 5C).

## 4. Discussion

The current cross sectional-study explored the relationship between two dominant bacteria genera (*Prevotella* and *Bacteroides*) and whole blood trace element levels among infertile patients and a healthy control group at the center of reproductive medicine, Shengjing Hospital of China Medical University. Consistent with previous studies, our results showed a significant reduction in the levels of zinc, magnesium, and iron among infertile patients. Meanwhile, the Log(*P/B*) in the infertile group was significantly increased. The obtained data demonstrated that Log(*P/B*) was negatively correlated with whole blood copper, zinc, magnesium, and iron levels, but not with calcium levels in infertile women. By comparing the quantity differences between *Prevotella*-enterotype and *Bacteroides*-enterotype among LL, NL, and HL of whole blood trace elements, we observed that only in whole blood iron did these two enterotypes differ significantly. In the LL of the whole blood iron population, *Prevotella*-enterotype seems more dominant; in turn, the number of *Bacteroides*-enterotype was higher in people with the HL of whole blood iron.

Currently, relevant studies have proved that human trace elements (Cu, Zn, Ca, Mg, Fe) are regulated by gut microbiota. *Thermotogae*, *Chlorobi*, *Lactobacillales*, and *Mollicutes* contain copper effluxers to protect against the harmful effects of Cu^2+^ and maintain copper homeostasis in the cytoplasm by controlling copper transport [30]. The composition of gut microbiota (especially colon microbiota [31]) affects zinc absorption. For example, there is a positive correlation between *Lactobacillaceae* and *Bifidobacterium spp.* with zinc [32]. Gut microbiota can promote Ca^2+^ uptake by regulating the function of Ca^2+^-carrying channel proteins, transient receptor potential vaniloid member 6 (TRPV6), which is located at the apex of intestinal cells, as well as Na^+^/Ca^2+^ exchanger (NCX1) and Ca^2+^ ATPase (PMCA1b), located at the base of intestinal cells [33]. Whisner et al. [34,35] revealed that the absorption of Ca^2+^ had a positive association with the genera *Oscillibacter*, *Bacteroides*, *Dialister*, and *Butyricicoccus*; those gut bacteria genera can ferment soluble corn fiber to produce short-chain fatty acids (SCFAs), reducing pH in the gut and promoting the solubility of calcium. Gut microbiota also assumes an important part in the bioavailability of Mg. Aljewitz et al. [36] and Bergillos-Meca et al. [37] revealed that *Lactobacillus* improves the availability of Mg. Fe^3+^ must be reduced to Fe^2+^ by iron reductase duodenal cytochrome b (DCYTB) before it can be absorbed by divalent metal transporter 1 in the small intestine [38]. Many gut microbes rely on high-affinity siderophores to absorb Fe^3+^, promoting iron absorption in the small intestine [39]. Some intestinal bacteria, for example, *Lactobacillus johnsonii* and *Lactobacillus reuteri,* can enhance cellular iron storage by involving in the inhibition of intestinal iron absorption pathways [40].

We found the significant differences in micronutrients (zinc, magnesium, iron), BMI, and education levels between the infertile and control groups. Several studies have shown a direct correlation between a higher BMI and a poorer fertility prognosis [41,42,43]. Zhao et al. [44] reported that the lower educational level was a risk factor for infertility. Previous research has shown that the levels of zinc, magnesium, and iron were significantly decreased in diseases associated with female infertility [45,46,47], which was consistent with our results, indicating that inadequate levels of micronutrients are related with unfavorable reproductive function [10]. Interestingly, in the infertile group, the levels of copper, zinc, magnesium, and iron were lower in the *Prevotella*-enterotype. Enterotypes were strongly associated with long-term diets, to be specific, *Bacteroides*-enterotype was associated with protein and animal fat while *Prevotella*-enterotype was associated with carbohydrates and fibres. People with *Prevotella*-enterotype eat less animal protein and fat (the main source of copper [48], iron [49], magnesium, and zinc) than those with *Bacteroides*-enterotype [19]. We speculate that different dietary habits and digestive abilities may be the reason for the different levels of trace elements between the two enterotypes.

Gut microbiota is considered to be an endocrine organ, which plays a significant role in female reproductive endocrine function. As a dominant bacterial genus in the intestinal tract, *Prevotella* is associated with female reproduction-related diseases. Comparing with the normal menstrual cycle (NMC) group, *Prevotella* was more abundant in women with irregular menstrual cycles (IMC) [50]. *Prevotella*, especially *Prevotella_9*, was positively correlated with total testosterone in PCOS [51,52]. The causal role of *Bacteroides* genus in reproduction-related diseases has not been convincingly proven. *Bacteroides vulgatus* was markedly elevated in the gut microbiota of PCOS individuals, accompanied by reduced levels of tauroursodeoxycholic acid and glycodeoxycholic acid [53]. However, On the phylum level, Jobira et al. reported that patients with PCOS had lower *Bacteroides* (*P* = 0.004) [54]. Another study showed that the proportion of *Bacteroides* in the gestational diabetes mellitus (GDM) group was significantly lower than that in the normoglycemic pregnant women (NOR) group [55]. Hence, the different trends of *Prevotella* and *Bacteroides* found in the women with reproductive diseases may be the reason why there were significant differences and associations between enterotypes and Log(*P*/*B*) with the trace elements only in the infertile group.

There is a close relationship between iron and reproductive function; meanwhile, our results demonstrated that *Prevotella*-enterotype and *Bacteroides*-enterotype are significantly associated with whole blood iron levels. We suspect that iron may contribute as an intermediary in the process of these two dominant genus and how they affect the reproductive function. *Prevotella* may impair reproductive function by reducing iron in the host, while *Bacteroides* may ameliorate impaired reproductive function by providing iron to the host.

The required trace metal elements needed by the human body mainly come from food. However, trace elements must be absorbed in the gastrointestinal tract to maintain an adequate supply of trace elements in blood and cellular stability. As reported by previous studies, iron deficiency in anemic infants showed higher *Prevotella* concentration than that in non-anemic infants [56,57]. In a study by Dillon et al. [58], *Prevotella copri* and *Prevotella stercorea* induced a higher fraction of IL-1β on colonic myeloid dendritic cells (mDCs) subsets CD1c^+^. IL-1β is a mucosal inflammatory cytokine that is a pathogenic contributor of anemia of inflammation in rheumatoid arthritis (RA) patients with lower iron and hemoglobin than in non-RA patients [59]. The results indicate that *Prevotella* may reduce iron levels by inducing IL-1β. High levels of iron can enhance serum cytokines and increase *Bacteroides* in feces, as analyzed by microbiome analysis [60]. After adding iron supplementation for 80 days to antibiotic-exposed mice, the composition of the dominant gut microbiota shifted to *Bacteroides* [61]. Wu et al. [11] reported that the long-term diet of people with *Bacteroides*-enterotype was more associated with protein and animal fat, which is an essential source of iron [49]. Macrophages regulate iron homeostasis in plasma by recycling and storing iron from senescent red blood cells and other damaged cells. This process was regulated by the interaction of heparin and ferroportin (FPN, SLC40A1), a multichannel plasma membrane protein that transfers excess iron from cells to blood plasma or extracellular fluid [62]. Vermet et al. [63] revealed that *Bacteroides fragilis*, a representative of the genus *Bacteroides*, lowered the serum iron levels by down-regulating FPN. This does not seem to agree with our results. Here are our inferences: (1) Serum/plasma trace element levels are mainly affected by recent diet, while whole blood trace element levels reflect long-term changes [64]; hence, further studies are needed to explore whether *Bacteroides fragilis* down-regulation of FPN can also reduce the iron content in the whole blood. (2) Vermet et al. emphasized that the effect of *Bacteroides fragilis* induced a decline in FPN on macrophages itself and goes beyond the regulation of iron homeostasis, mainly seen in systemic iron deficiency [65,66] or infection with certain pathogens [63].

We observed that the *Prevotella*-enterotype, especially when Log(*P/B*) > −0.27, has a predictive power and an acceptable sensitivity and specificity of deficient whole blood iron levels in the obesity group. As reviewed by Zhao et al. [67], obesity is significantly associated with iron deficiency. Obesity influences iron metabolism by affecting iron absorption, storage, transport, utilization, recycling, and homeostasis regulation [68]. Some *Prevotella* strains had pathobiontic properties, which promoted diseases such as obesity and metabolic syndrome [69]. Moreover, the genera *Prevotella* and *Collinsella* were more prevalent in obese adolescents [70]. Obesity can negatively impact on women’s fertility and success with treatment, and significant maternal and perinatal morbidity and mortality [71]. These studies suggested that the relationship between *Prevotella* and iron is more relevant in female reproduction compared with non-obesity people.

However, it must be admitted that our research has certain limitations. We compared the living habits, eating habits, and medication history of the patients, and found that the differences were not significant. However, the pathological and gut microbiota genetic heterogeneity in different enrolled participants may become confounding factors in our study. Although whole blood trace elements can well reflect the status of trace elements in the human body and the detection method is accurate and convenient, in the future, we still need to analyze trace elements in other parts. Meanwhile, we will carry out a multi-center study for enterotypes and trace element analysis, hoping to make our results more generalized.

## 5. Conclusions

In summary, our study revealed the correlation between Log(*P/B*) and whole blood trace elements, especially with whole blood iron in female infertility. *Prevotella*-enterotype is associated with LL of whole blood iron, while *Bacteroides*-enterotype is associated with HL of whole blood iron. These preliminary results opened new horizons toward improving our understanding of the biological role of enterotypes in trace element metabolism, highlighting the enterotypes as acceptable and promising biomarkers in personalized nutrition for women with reproductive needs. Combined with a previous study [72], we will conduct intervention trials in the future to further explore the role of gut microbiota in the metabolism, absorption, and utilization of trace elements.

## Figures and Tables

**Table 1:** Baseline characteristics of the study population between infertile group and healthy control group.

Characteristics	Infertile Group	Control Group	*P* Value
**Baseline**			
N (%)	469 (72.04)	182 (27.96)	
Log(*P/B*)	−0.32 (−3.45, 0.53)	−2.86 (−3.76, 0.03)	<0.001
Age (years)	33.04 ± 4.29	33.32 ± 4.37	0.459
BMI (kg/m^2^)	23.68 ± 2.80	22.69 ± 4.09	0.001
Current smoking, N (%)	14 (2.99)	3 (1.65)	0.423
**Trace elements**			
Cu (μmol/L)	15.89 ± 3.95	16.25 ± 3.75	0.083
Zn (μmol/L)	94.07 ± 12.41	97.59 ± 11.55	0.001
Ca (μmol/L)	1.67 ± 0.17	1.65 ± 0.18	0.206
Mg (μmol/L)	1.46 ± 0.14	1.50 ± 0.13	<0.001
Fe (μmol/L)	8.39 ± 0.71	8.84 ± 0.56	<0.001
**Education, N (%)**			<0.001
Middle school and below	68 (10.45)	13 (1.20)	
High school	84 (12.90)	17 (2.61)	
College	238 (36.56)	97 (14.90)	
Master and above	79 (12.14)	55 (9.24)	
**Diagnosis, N (%)**			
RSA	114 (24.31)	−	
PCOS	81 (17.27)	−	
RIF	43 (9.17)	−	
DOR	17 (3.62)	−	
Tubal factor infertility/endometriosis	128 (27.29)	−	
Unexplained infertility	86 (18.34)	−	
**Childbirth and abortion history**			
Previous pregnancies	93	58	
Previous deliveries	74	20	
Previous spontaneous abortions	101	9	

*P*-enterotype, *Prevotella*-enterotype; *B*-enterotype, *Bacteroides*-enterotype; Cu, copper; Zn, zinc; Ca, calcium; Mg, magnesium; Fe, iron; RSA, recurrent spontaneous abortion; PCOS, polycystic ovarian syndrome; RIF, repeated implantation failure; DOR, diminished ovarian reserve. Mean ± standard deviation or median (interquartile range) are shown. The Student’s t test was used for continuous variables; the Chi-square test and Fisher’s exact test were used for categorical variables.

**Table 2:** Whole blood trace element levels of the study population according to enterotypes in healthy and infertile group.

	Control Group (n = 182)			Infertile Group (n = 469)		
	*P*-Enterotype	*B*-Enterotype	*P* Value	*P*-Enterotype	*B*-Enterotype	*P* Value
N	106	76		220	249	
Cu (μmol/L)	15.70 ± 3.66	16.33 ± 3.87	0.262	15.48 ± 4.28	16.26 ± 3.59	0.033
Zn (μmol/L)	97.42 ± 12.44	97.83 ± 10.27	0.815	91.71 ± 11.99	96.16 ± 12.41	<0.001
Ca (μmol/L)	1.66 ± 0.20	1.64 ± 0.15	0.441	1.67 ± 0.19	1.66 ± 0.16	0.549
Mg (μmol/L)	1.50 ± 0.13	1.51 ± 0.13	0.586	1.42 ± 0.12	1.49 ± 0.14	<0.001
Fe (μmol/L)	8.82 ± 0.56	8.86 ± 0.57	0.591	8.17 ± 0.64	8.58 ± 0.71	<0.001

*P*-enterotype, *Prevotella*-enterotype; *B*-enterotype, *Bacteroides*-enterotype; Cu, copper; Zn, zinc; Ca, calcium; Mg, magnesium; Fe, iron. Mean ± standard deviations are shown. The Student’s t test was used for continuous variables.

**Table 3:** Comparison of *Prevotella*-enterotype and *Bacteroides*-enterotype in the LL, NL, and HL of the five whole blood trace elements among infertile group.

Trace Element	Status	Concentration (µmol/L)	*P*-Enterotype (N)	*B*-Enterotype (N)	χ2	*P* Value
Cu	LL	<9.25	15	8	3.300	0.187
	NL	9.25–21.21	193	228		
	HL	>21.21	12	13		
Zn	LL	<74.66	11	12	5.420	0.063
	NL	74.66-111.17	204	220		
	HL	>111.17	5	17		
Ca	LL	<1.42	6	9	3.168	0.186
	NL	1.42–1.90	202	234		
	HL	>1.90	12	6		
Mg	LL	<1.25	6	6	4.048	0.140
	NL	1.25-1.70	209	228		
	HL	>1.70	5	15		
Fe	LL	<7.37	14	9	13.309	0.001
	NL	7.37–9.60	203	220		
	HL	>9.60	3	20		

*P*-enterotype, *Prevotella*-enterotype; *B*-enterotype, *Bacteroides*-enterotype; Cu, copper; Zn, zinc; Ca, calcium; Mg, magnesium; Fe, iron; LL, low level group; NL, normal level group; HL, high level group. The Chi-square test and Fisher’s exact test were used to compare categorical variables.

**Table 4:** Description of the diet and baseline information in infertile participants categorized by whole blood iron status.

Characteristic	Whole Blood Iron Status			*P* Value ^a^	*P* Value ^b^	*P* Value
	LL	NL	HL			
N (%)	23	423	23			
Log(*P*/*B*)	−0.57 (−3.15, 0.36)	−2.65 (−3.77, 0.03)	−3.71 (−4.05, −3.06)	0.013	0.006	0.002
Age (years)	33.00 (30.00, 34.00)	33.00 (31.00, 36.00)	32.00 (30.00, 36.00)	0.669	0.606	0.998
BMI (kg/m^2^)	23.59 (20.57, 24.80)	23.80(22.00, 26.50)	23.20 (21.17, 26.38)	0.043	0.482	0.045
Diet in the last month (N)				0.134	0.325	0.184
A vegetarian diet	2	10	0			
A meat-based diet	1	17	2			
A meat and vegetarian diet	20	396	21			
Drinking in the last month (N)				0.945	0.751	0.953
None	21	386	22			
≤3 times a month	2	35	1			
>3 times a month	0	2	0			

LL, low level group; NL, normal level group; HL, high level group; BMI, body mass index. Log(*P/B*), Log (*Prevotella*/*Bacteroides*). Median (interquartile range) is shown. The Kruskal‒Wallis test was conducted for continuous variables, and the Chi-square test and Fisher’s exact test were conducted for categorical variables. ^a^ Comparing the LL and NL of whole blood iron after the post-hoc test. ^b^ Comparing the HL and NL of whole blood iron after the post-hoc test.

**Table 5:** Predictive Log(*P/B*) performance in LL of whole blood iron among infertile lean/normal, overweight, and obese patients, respectively.

	AUC	SE	Cutoff	95% CI	Sensitivity %	Specificity %	*P* Value
Lean/normal	0.594	0.054	−0.64	0.487–0.700	84.2	61.3	0.172
Overweight	0.599	0.073	−3.23	0.454–0.743	51.1	96.4	0.633
Obesity	0.894	0.087	−0.27	0.724–0.993	98.8	78.8	0.042

AUC, the area under the curve; SE, standard error; CI, confidence interval.

**Table 6:** Predictive Log(*P/B*) performance in HL of whole blood iron among infertile lean/normal, overweight, and obese patients, respectively.

	AUC	SE	Cutoff	95% CI	Sensitivity %	Specificity %	P Value
Lean/normal	0.648	0.052	−2.76	0.546–0.750	55.7	82.4	0.041
Overweight	0.863	0.040	−3.99	0.786–0.941	78.1	92.7	0.013
Obesity	0.818	0.088	−3.70	0.645–0.992	72.7	78.3	0.136

AUC, the area under the curve; SE, standard error; CI, confidence interval.

## Data Availability

The data used to support the findings of this study are available from the corresponding author upon request.

## Supplementary Materials

The following supporting information can be downloaded at: https://www.mdpi.com/article/10.3390/nu14153195/s1, Table S1: Comparison of *Prevotella*-enterotype and *Bacteroides*-enterotype in the LL, NL, and HL of the five whole blood trace elements among healthy control group; Table S2: Description of the diet and baseline information in healthy control group categorized by whole blood iron status; Table S3: Baseline characteristics of the study population according to enterotypes among RSA group.; Table S4: Baseline characteristics of the study population according to enterotypes among PCOS group; Table S5: Baseline characteristics of the study population according to enterotypes among RIF group; Table S6: Baseline characteristics of the study population according to enterotypes among DOR group; Table S7: Baseline characteristics of the study population according to enterotypes among tubal factor infertility/endometriosis group; Table S8: Baseline characteristics of the study population according to enterotypes among unexplained infertility group; Table S9: The primer and TaqMan probe sequences.
