# Supplementary material for: Association of Gut Microbiota Enterotypes with Blood Trace Elements in Women with Infertility
Source: Nutrients. 2022 Aug 4;14(15):3195. doi: 10.3390/nu14153195 (PMC9370633; doi:10.3390/nu14153195)
Supplement: Supplementary file 1 [file nutrients-14-03195-s001.zip › nutrients-1806494-supplementary.pdf]

Supplemental Table S1. Comparison of *Prevotella*-enterotype and *Bacteroides*-enterotype in the LL, NL and HL of the five whole blood trace elements among healthy control group.

Trace Element	Status	Concentration (μmol/L)	*P*-enterotype (N)	*B*-enterotype (N)	χ2	*P* value
Cu	LL	＜9.28	6	3	2.532	0.281
	NL	9.28-21.81	97	67		
	HL	＞21.81	3	6		
Zn	LL	＜75.76	7	2	2.867	0.238
	NL	75.76-114.62	92	72		
	HL	＞114.62	7	2		
Ca	LL	＜1.42	6	2	0.980	0.721
	NL	1.42-1.92	95	71		
	HL	＞1.92	5	3		
Mg	LL	＜1.25	4	3	1.654	0.498
	NL	1.25–1.69	100	69		
	HL	＞1.69	2	4		
Fe	LL	＜7.92	3	4	0.885	0.724
	NL	7.92-9.82	98	68		
	HL	＞9.82	5	4		

P-enterotype, *Prevotella*-enterotype; *B*-enterotype, *Bacteroides*-enterotype; Cu, copper; Zn, zinc; Ca, calcium; Mg, magnesium; Fe, iron; LL, low levels group; NL, normal levels group; HL, high levels group. The Chi-square test and Fisher's exact test were used to compare categorical variables.

Supplemental Table S2. Description of the diet and baseline information in healthy control group categorized by whole blood iron status.

**Characteristic**	**Whole blood iron status**					
	**LL**	**NL**	**HL**	***P* value**[a]	***P* value**[b]	***P* value**
N (%)	7	166	9			
Log(*P*/*B*)	-0.25 (-3.39, 0.57)	-2.75 (-3.78, 0.06)	-2.00 (-3.89, -0.18)	0.497	0.764	0.478
Age (year)	34.00 (32.00, 34.00)	33.00 (30.00, 36.00)	34.00 (29.00,34.00)	0.999	0.624	0.686
BMI (kg/m^2)	20.80 (18.1, 24.70)	22.30 (19.80, 25.40)	21.70 (20.25, 24.70)	0.536	0.911	0.575
Diet in the last month (N)				0.134	0.085	0.053
A vegetarian diet	0	5	1			
A meat-based diet	1	3	1			
A meat and vegetarian diet	6	158	7			
Drinking in the last month (N)				0.630	0.087	0.149
None	7	137	8			
≤3 times a month	0	27	0			
＞3 times a month	0	2	1			

LL, low levels group; NL, normal levels group; HL, high levels group; BMI, body mass index. Log(P/B), Log (*Prevotella*/*Bacteroides*). Median (interquartile range) is shown. The Kruskal–Wallis test was conducted for continuous variables, and the Chi-square test and Fisher's exact test were conducted for categorical variables.

[a]Comparing the LL and NL of whole blood iron after the post-hoc test.

[b]Comparing the HL and NL of whole blood iron after the post-hoc test.

Supplemental Table S3. Baseline characteristics of the study population according to enterotypes among RSA group.

	P-enterotype	*B*-enterotype	*P* value
Cu (μmol/L)	14.96±3.00	16.59±3.91	**0.027**
Zn (μmol/L)	90.04±8.71	95.33±12.42	0.059
Ca (μmol/L)	1.66±0.15	1.68±0.14	0.668
Mg (μmol/L)	1.46±0.14	1.48±0.13	0.440
Fe (μmol/L)	8.28±0.60	8.36±0.45	**0.048**

RSA, recurrent spontaneous abortion; *P*-enterotype, *Prevotella*-enterotype; *B*-enterotype, *Bacteroides*-enterotype; Cu, copper; Zn, zinc; Ca, calcium; Mg, magnesium; Fe, iron; Mean ± standard deviation is shown. The Student's t test was used for continuous variables.

Supplemental Table S4. Baseline characteristics of the study population according to enterotypes among PCOS group.

	P-enterotype	*B*-enterotype	*P* value
Cu (μmol/L)	15.56±2.88	16.34±3.59	0.437
Zn (μmol/L)	90.61±11.41	96.68±10.59	**0.029**
Ca (μmol/L)	1.71±0.25	1.63±0.14	0.148
Mg (μmol/L)	1.50±0.13	1.51±0.11	0.879
Fe (μmol/L)	8.35±0.46	8.41±0.49	0.689

PCOS, polycystic ovarian syndrome; *P*-enterotype, *Prevotella*-enterotype; *B*-enterotype, *Bacteroides*-enterotype; Cu, copper; Zn, zinc; Ca, calcium; Mg, magnesium; Fe, iron; Mean ± standard deviation is shown. The Student's t test was used for continuous variables.

Supplemental Table S5. Baseline characteristics of the study population according to enterotypes among RIF group.

	P-enterotype	*B*-enterotype	*P* value
Cu (μmol/L)	15.43±2.77	16.52±3.75	0.576
Zn (μmol/L)	92.27±11.30	93.96±10.97	0.636
Ca (μmol/L)	1.63±0.13	1.66±0.11	0.484
Mg (μmol/L)	1.45±0.11	1.49±0.12	0.245
Fe (μmol/L)	8.19±0.60	8.49±0.46	0.255

RIF, repeated implantation failure; *P*-enterotype, *Prevotella*-enterotype; *B*-enterotype, *Bacteroides*-enterotype; Cu, copper; Zn, zinc; Ca, calcium; Mg, magnesium; Fe, iron; Mean ± standard deviation is shown. The Student's t test was used for continuous variables.

Supplemental Table S6. Baseline characteristics of the study population according to enterotypes among DOR group.

	P-enterotype	*B*-enterotype	*P* value
Cu (μmol/L)	14.74±3.01	15.81±2.73	0.543
Zn (μmol/L)	91.29±9.15	97.61±10.61	**0.036**
Ca (μmol/L)	1.68±0.12	1.71±0.14	0.751

Mg (μmol/L)	1.42±0.06	1.48±0.15	**0.023**
Fe (μmol/L)	7.80±0.40	8.53±0.23	**0.004**

DOR, diminished ovarian reserve; *P*-enterotype, *Prevotella*-enterotype; *B*-enterotype, *Bacteroides*-enterotype; Cu, copper; Zn, zinc; Ca, calcium; Mg, magnesium; Fe, iron; Mean ± standard deviation is shown. The Student's t test was used for continuous variables.

Supplemental Table S7. Baseline characteristics of the study population according to enterotypes among tubal factor infertility/endometriosis group.

	P-enterotype	*B*-enterotype	*P* value
Cu (μmol/L)	14.15±4.15	16.26±3.47	0.154
Zn (μmol/L)	93.48±11.82	99.78±10.13	**0.042**
Ca (μmol/L)	1.60±0.14	1.70±0.16	0.462
Mg (μmol/L)	1.43±0.06	1.44±0.11	0.813
Fe (μmol/L)	7.99±0.46	8.94±0.50	**0.011**

P-enterotype, *Prevotella*-enterotype; *B*-enterotype, *Bacteroides*-enterotype; Cu, copper; Zn, zinc; Ca, calcium; Mg, magnesium; Fe, iron; Mean ± standard deviation is shown. The Student's t test was used for continuous variables.

Supplemental Table S8. Baseline characteristics of the study population according to enterotypes among unexplained infertility group.

	P-enterotype	*B*-enterotype	*P* value
Cu (μmol/L)	14.25±32.95	16.10±3.71	0.337
Zn (μmol/L)	90.27±8.17	94.90±9.59	0.332
Ca (μmol/L)	1.64±0.09	1.66±0.14	0.824
Mg (μmol/L)	1.45±0.08	1.47±0.12	0.637
Fe (μmol/L)	8.06±0.31	8.27±0.57	0.450

P-enterotype, *Prevotella*-enterotype; *B*-enterotype, *Bacteroides*-enterotype; Cu, copper; Zn, zinc; Ca, calcium; Mg, magnesium; Fe, iron; Mean ± standard deviation is shown. The Student's t test was used for continuous variables.

Supplemental Table S9. The primer and TaqMan probe sequences

		Sequence
Prevotella	Forward Primer	CCAGCCAAGTAGCGTGCA
	Reverse Primer	TGGACCTTCCGTATTACCGC
Bacteroides	Forward Primer	VGATGGGGATGCGTTCCATTAG
	Reverse Primer	CATCCTTCACGCTACTTGGCTGG
